# “Of mice and men”: the relevance of Cometin and Erythropoietin origin for its effects on murine spiral ganglion neuron survival and neurite outgrowth *in vitro*

**DOI:** 10.3389/fnins.2023.1224463

**Published:** 2023-08-10

**Authors:** Jana Schwieger, Ziwen Gao, Thomas Lenarz, Gordon Munro, Kenneth A. Petersen, Verena Scheper

**Affiliations:** ^1^Department of Otorhinolaryngology, Hannover Medical School, Hannover, Germany; ^2^Lower Saxony Center for Biomedical Engineering, Implant Research and Development (NIFE), Hannover, Germany; ^3^Cluster of Excellence "Hearing4all" EXC 1077/2, Hannover, Germany; ^4^Ear Nose and Throat Institute and Department of Otorhinolaryngology, Eye & ENT Hospital, Fudan University, Shanghai, China; ^5^Hoba Therapeutics ApS, Copenhagen, Denmark

**Keywords:** pharmacotherapy, inner ear, cochlea, neuroprotection, species specific, neurite outgrowth, drug delivery

## Abstract

Neurotrophic factors (NTF) play key roles in the survival of neurons, making them promising candidates for therapy of neurodegenerative diseases. In the case of the inner ear, sensorineural hearing loss (SNHL) is characterized over time by a degeneration of the primary auditory neurons, the spiral ganglion neurons (SGN). It is well known that selected NTF can protect SGN from degeneration, which positively influences the outcome of cochlear implants, the treatment of choice for patients with profound to severe SNHL. However, the outcome of studies investigating protective effects of NTF on auditory neurons are in some cases of high variability. We hypothesize that the factor origin may be one aspect that affects the neuroprotective potential. The aim of this study was to investigate the neuroprotective potential of *human* and *mouse* Erythropoietin (EPO) and Cometin on rat SGN. SGN were isolated from neonatal rats (P 2–5) and cultured in serum-free medium. EPO and Cometin of mouse and human origin were added in concentrations of 0.1, 1, and 10 ng/mL and 0.1, 1, and 10 μg/mL, respectively. The SGN survival rate and morphology, and the neurite outgrowth were determined and compared to negative (no additives) and positive (brain-derived neurotrophic factor, BDNF) controls. A neuroprotective effect of 10 μg/mL human Cometin comparable to that obtained with BDNF was observed in the SGN-culture. In contrast, mouse Cometin was ineffective. A similar influence of 10 μg/mL human and mouse and 1 μg/mL human Cometin on the length of regenerated neurites compared to BDNF was also detected. No other Cometin-conditions, and none of the EPO-conditions tested had neuroprotective or neuritogenic effects or influenced the neuronal morphology of the SGN. The neuroprotective effect of 10 μg/mL human Cometin on SGN indicates it is a potentially interesting protein for the supportive treatment of inner ear disorders. The finding that mouse Cometin had no effect on the SGN in the parallel-performed experiments underlines the importance of species origin of molecules being screened for therapeutic purpose.

## 1. Introduction

Over 5% of the world’s population has disabling hearing loss. It is estimated that by 2050 over 900 million people will have disabling hearing loss (WHO | Deafness and hearing loss, 2021). Sensorineural hearing loss (SNHL) results from damage and degeneration of the cochlear sensory cells that subsequently fail in neurotransmitter release and initiation of action potential generation. Cochlear implants (CI) electrically stimulate the primary auditory neurons, the spiral ganglion neurons (SGN), and evoke a hearing sensation. It is widely assumed that the condition of the auditory nerve plays a significant role in determining the quality of information transferred from the CI to the brain (Lenarz, 2017). Under healthy conditions, SGN receive support from neurotrophic factors (NTF) released from supporting cells, sensory hair cells of the organ of Corti and target neurons within the cochlear nucleus (Rubel and Fritzsch, 2002; Roehm and Hansen, 2005; Green et al., 2012). The loss of the sensory epithelium in SNHL leads to insufficient neurotrophic support of the neurons, which subsequently degenerate over time (Versnel et al., 2007; Zilberstein et al., 2012) finally resulting in reduced CI performance (Lenarz, 2017). Accordingly, NTF such as brain-derived neurotrophic factor (BDNF), or glial-cell line derived neurotrophic factor (GDNF), have been shown to prevent SGN and their peripheral and central processes from degeneration following SNHL *in vivo* (Scheper et al., 2009, 2020; Havenith et al., 2011; Leake et al., 2011; Vink et al., 2020).

For Erythropoietin (EPO) neuroprotective effects in preclinical models of acute and chronic degenerative disorders (Ercan et al., 2018) and improved neuronal survival *per se* (Bartesaghi et al., 2005; Wan et al., 2022) have also been described. EPO is the principal regulator of hematopoiesis mediated by its ability to inhibit apoptosis and stimulate proliferation and differentiation of erythroid precursor cells (Bartesaghi et al., 2005). For SGN therapy, the effects of EPO treatment are highly varied. There are reports that EPO treatment (i) decreases spontaneous apoptosis of cultured murine SGN (Zhong et al., 2018), (ii) results in significantly less SGN loss in an animal model of SNHL (Bächinger et al., 2018), (iii) has no positive effect on SGN survival but promotes neurite outgrowth (Berkingali et al., 2008) or (iv) has no effect on survival or neurite outgrowth after administration (Kaiser et al., 2013). Additionally, in clinical trials on neurodegenerative disease, EPO often fails to be protective as recently reviewed by Vittori et al. (2021). We hypothesize that the variation in neuroprotective effects might partially be due to the use of EPO from different species for the experiments.

Within this study, we aimed to investigate whether the species from which putative therapeutic proteins are derived, in this case mouse vs. human exert different effects on SGN *in vitro*. In addition to EPO we also tested the SGN supportive effects of Cometin (also known as Meteorin-like or Metrnl) obtained from different species. Cometin is a secreted neurotrophic protein with tissue specific expression (Jørgensen et al., 2012; Zheng et al., 2016). It has previously been demonstrated that Cometin supports neuronal survival and electrical responsiveness in the deafened inner ear *in vivo* (Jørgensen et al., 2012).This is the first study investigating the effects of human and mouse Cometin on SGN *in vitro*.

## 2. Materials and methods

### 2.1. SGC preparation and dissociation

All experiments were performed in accordance with the German “Law on Protecting Animals” and with the European Directive 86/609/EEC of the European Communities Council for protection of animals used for experimental purposes and registered with the local authorities of Hannover Medical School. Due to its common use as *in vitro* assay for first screening of inner ear therapeutics and to keep the number of animals used for the experiments as low as possible, the dissociated neonatal SGN-culture was performed (Whitlon, 2017; Bertram et al., 2019; Szobota et al., 2019; Sun et al., 2020). Neonatal Sprague–Dawley rats of both sexes (2–5 days old) were dissected for spiral ganglion cell (SGC) harvesting. The detailed preparation and dissociation procedure have been published previously (Schwieger et al., 2015). In short, rats were decapitated, temporal bones harvested, the spiral ganglia separated and transferred to ice-cold Ca^2+^/Mg^2+^ free Hank’s balanced salt solution (HBSS) (Gibco Invitrogen, Karlsruhe, Germany).

The enzymatic and mechanical dissociation of the SGC was performed using 0.1% trypsin (Biochrom, Berlin, Germany) and 0.01% DNase I (30–40 ganglions/2 mL, Roche, Mannheim, Germany). Enzymatic dissociation was stopped by withdrawal of the supernatant and addition of fetal calf serum (FCS, Invitrogen, Karlsruhe, Germany) and the SGC were mechanically dissociated in 1 mL of the serum-free medium (see below). Trituration was repeated until no cell clusters were visible in the suspension. The cell number was counted using the trypan blue (Sigma–Aldrich, Taufkirchen, Germany) exclusion test in a Neubauer chamber (BRAND GmbH, Wertheim, Germany). Finally, the cells were seeded in poly-DL-ornithine (0.1 mg/mL; Sigma Aldrich, Taufkirchen, Germany)/laminin (0.01 mg/mL, Naturel Mouse Laminin; Invitrogen, Karlsruhe, Germany) coated 96-multiwell culture plates (Nunc A/S, Roskilde, Denmark).

### 2.2. SGC cultivation

Cells were seeded in a concentration of 1 × 10^4^ cells/well. The serum-free culture medium consisted of Panserin 401 (PAN BIOTECH, Passau, Germany) supplemented with Hepes-buffer solution (23.4 μmol/mL, Invitrogen, Carlsbad, United States), PBS (0.172 mg/mL; Gibco, Thermo Fisher Scientific, Waltham, United States), penicillin (30 U/mL; Biochrom, Berlin, Germany), glucose (0.15%; Braun AG, Melsungen, Germany), insulin (8.7 μg/mL; Biochrom, Berlin, Germany) and N2-supplement (0.1%, Invitrogen, Carlsbad, CA, United States). Culture was performed in an incubator (Binder, Tuttlingen, Germany) in a humidified atmosphere with 5% CO_2_ at 37°C.

Each experimental condition (Table 1) was performed in triplicate in three independent experiments. Mouse and human EPO (Mouse: ABIN2720509, Human: ABIN2017771, antibodies-online GmbH, Aachen, Germany), as well as mouse and human Cometin (Mouse: Cat. # 6679-MN, Human: Cat. # 9339-MN, R&D Systems, United States) were added in concentrations of 0.1, 1, and 10 ng/mL (EPO) and 0.1, 1, and 10 μg/mL (Cometin) to the SGC culture at the time of seeding. A seeding control giving the number of initially present SGN was included in each experiment. SGC cultivated in control media with addition of 50 ng/mL BDNF (human recombinant BDNF, from *E. coli*; Invitrogen) served as positive control (PC) (Wefstaedt et al., 2005; Schwieger et al., 2015, 2020), while the negative control (NC) contained no additives. Cell fixation was performed with 1:1 acetone/methanol solution after 4 h of cell cultivation in case of the seeding control and after 48 h for all other conditions.

**Table 1:** Summary of treatment groups.

Treatment group		Description
SC		Seeding control, no additives
NC		Negative control, no additives
PC		Positive control, 50 ng/mL BDNF
MC	MC 0.1	Mouse Cometin 0.1 μg/mL
	MC 1	Mouse Cometin 1 μg/mL
	MC 10	Mouse Cometin 10 μg/mL
HC	HC 0.1	Human Cometin 0.1 μg/mL
	HC 1	Human Cometin 1 μg/mL
	HC 10	Human Cometin 10 μg/mL
ME	ME 0.1	Mouse EPO 0.1 ng/mL
	ME 1	Mouse EPO 1 ng/mL
	ME 10	Mouse EPO 10 ng/mL
HE	HE 0.1	Human EPO 0.1 ng/mL
	HE 1	Human EPO 1 ng/mL
	HE 10	Human EPO 10 ng/mL

### 2.3. Immunocytochemistry

Cells were immunocytochemically stained with monoclonal antibody against Neurofilament 200 kD to distinguish neurons from the non-neuronal cells. A detailed protocol has been described previously (Wefstaedt et al., 2005; Schwieger et al., 2016). Briefly summarized, after fixation, cells were rinsed in PBS three times followed by blocking of non-specific binding sites with horse serum. Cells were incubated with monoclonal Neurofilament 200 kD primary antibody in antibody dilution buffer (1:100) and then the secondary antibody biotinylated horse anti-mouse IgG (1:2000) was added. DAB (3′-diaminobenzidine) labeling was performed in accordance with the staining kit protocol to produce brown precipitate where secondary antibody was present. Used antibodies and kits are listed in Table 2. Cell counts and images of the stained cells were performed with an inverse microscope (Axio; Carl Zeiss, Jena, Germany/Keyence BioRevoBZ-9000E + BZ II Viewer; Keyence, Osaka, Japan) and the cellSense/BZ-BZ II Analyzer software (Olympus Life Science; Keyence) manually.

**Table 2:** Antibodies and kits used for the staining of SGN.

Antibody	Antigen	Host, type, dilution	Company, cat.-no
Primary antibodies:			
Monoclonal antibody Neurofilament 200 kD	200 kD Neurofilament	Mouse, monoclonal, 1:500	Novocastra, Leica Biosystems; #NCL-L-NF200-N52
Secondary antibodies:			
Biotinylated horse anti-mouse IgG + DAB	Mouse IgG–H&L	Horse, 1:2000	Vectastain Elite ABC-kit + DAB Substrate Kit, Peroxidase, Vector Laboratories; #PK-6100 + #SK-4100

### 2.4. SGN analysis

#### 2.4.1. SGN survival rate

Surviving SGN were defined as cells stained positive for neurofilament and with neurites at least three times longer than the soma diameter (Gillespie et al., 2001) and were counted per well to determine the number of surviving neurons. This SGN number was subsequently divided by the number of SGN initially present (seeding control) and multiplied by 100 to calculate the neuronal survival rate in percentage.

#### 2.4.2. Neuronal morphology

**Figure 1:**
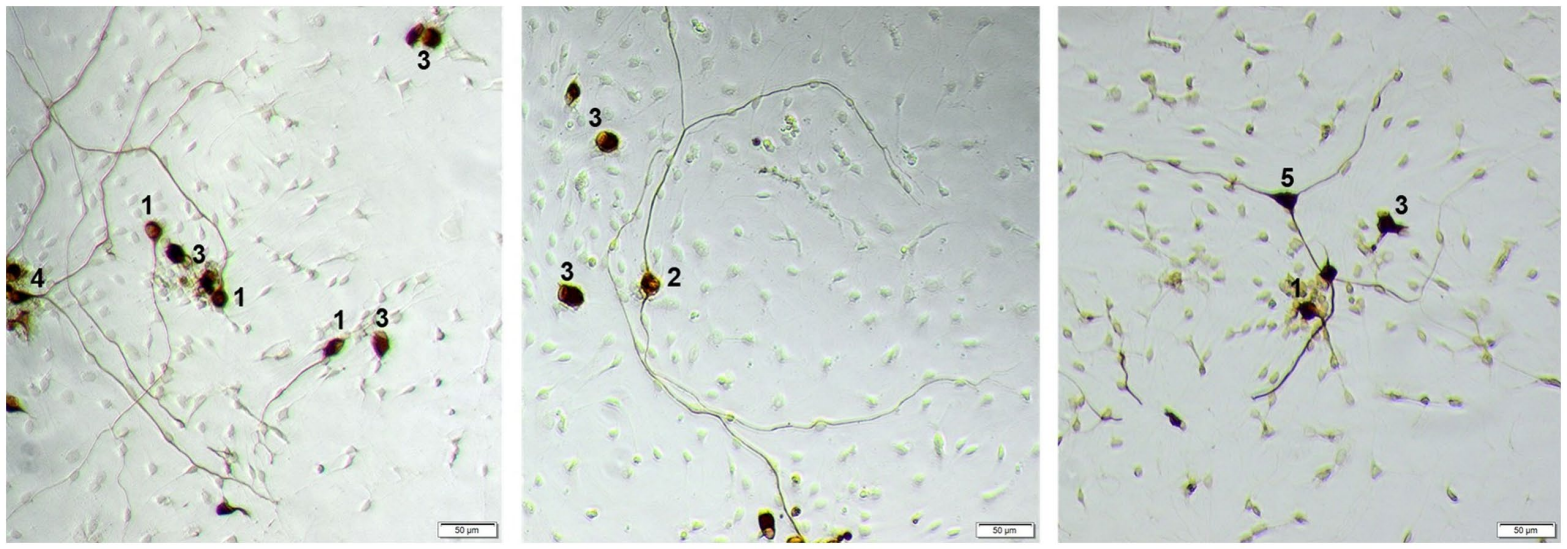
Example of different morphologies of SGN. 1: monopolar neuron; 2: bipolar neuron; 3: neuron with no neurites, 4: pseudomonopolar neuron; 5: multipolar neuron. Neurons are stained against neurofilament with DAB, contrast of the images is digitally improved. Scale bar: 50 μm.

According to Whitlon et al. (2007), SGN were classified as follows: monopolar (one neurite), bipolar (two neurites), multipolar (> two neurites), pseudomonopolar (branching of neurite within one soma diameter) and no neurites (Whitlon et al., 2007; Figure 1).

#### 2.4.3. Neurite length measurement

For the analysis of the SGN neurite length, images were taken from at least five fields of view evenly distributed over the well area (Schwieger et al., 2015) and processed using an image analysis program.^1^ The 15 longest neurites in these five fields of view (3 neurites/field) were measured using the polygon function of the image analysis program. For wells with less than 15 neurons in total, the longest neurite of each neuron was measured. If one cell presented several neurites, the longest neurite was selected for analysis.

### 2.5. Statistical analysis

Statistical analysis was performed using GraphPad Prism® software 9. Data were checked for normal distribution using the D’Agostino and Pearson normality test. Data from neuronal survival rate and morphology passed normality test and were analyzed using one-way ANOVA followed by Tukey’s Multiple Comparison Test. The measured neurite length were not normally distributed and Kruskal Wallis test followed by Dunn’s Multiple Comparison Test was performed. The data are reported as mean ± standard error of the mean (SEM). Statistical significance was considered at *p* values less than 0.05.

## 3. Results

### 3.1. Neuronal survival

**Figure 2:**
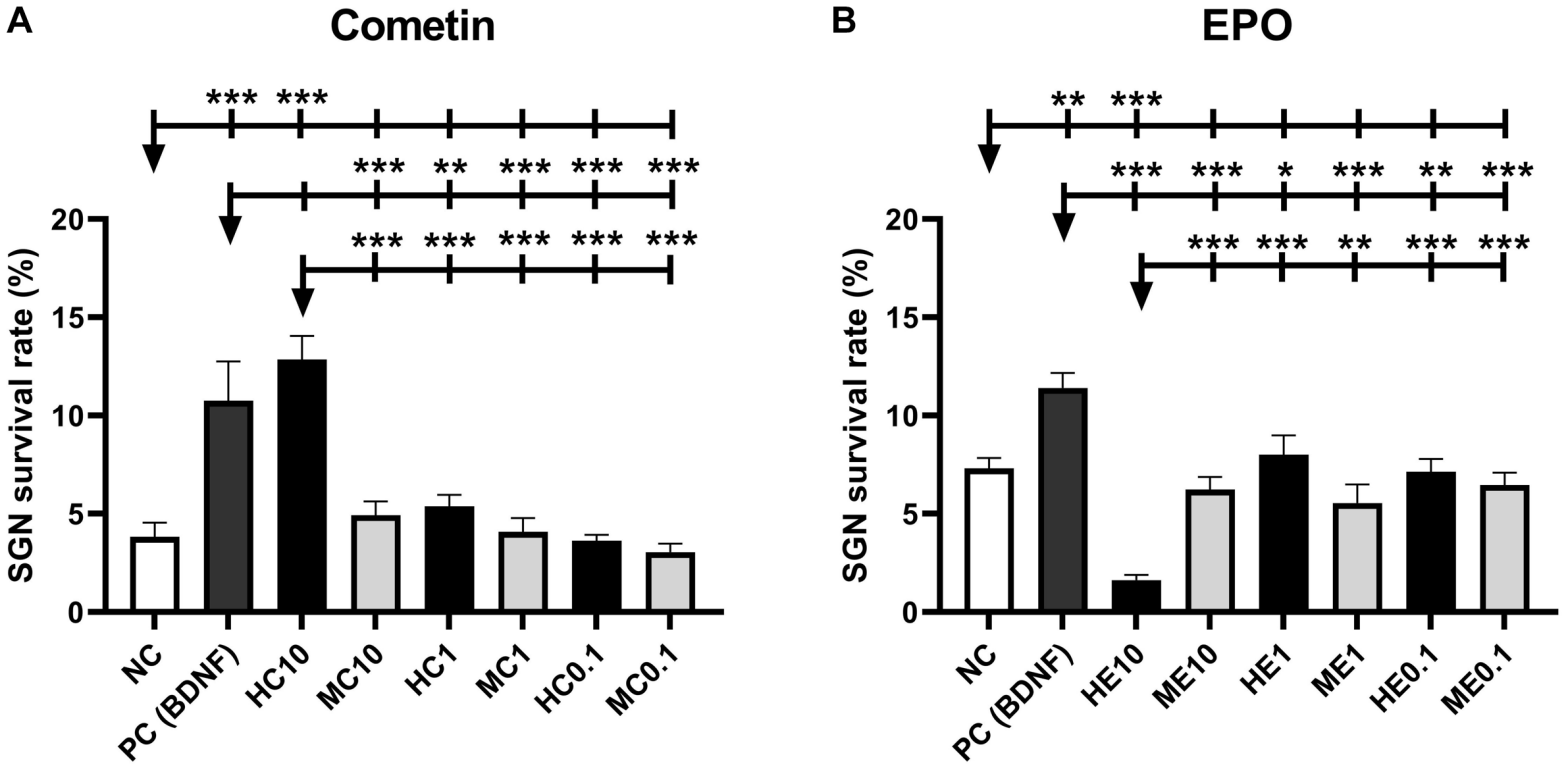
SGN survival rate after treatment with mouse and human Cometin **(A)** or EPO **(B)**. No factor-application served as negative control (NC) and 10 ng/mL BDNF served as a positive control (PC). BDNF increased neuronal survival in both test series significantly. 10 μg/mL human Cometin significantly increased the SGN survival rate compared to the NC and all other Cometin conditions and did not differ from PC. In contrast, the addition of 10 ng/mL human EPO significantly decreased SGN survival compared to NC, PC, and the other EPO conditions. No other treatment affected the SGN survival rate significantly compared to the NC. While 10 μg/mL human Cometin significantly improved SGN survival compared to the same concentration of mouse Cometin and the lower human concentrations, the opposite was the case for EPO. Here the 10 ng/mL of human EPO significantly reduced the SGN survival compared to the mouse EPO of the same concentration and the tested lower human concentrations. Asterisks above the error bars indicate the significant differences between the marked experimental conditions and the respective group indicated by the arrowhead. *p < 0.05, **p < 0.01, ***p < 0.001; independent experiments: 3; wells per experiment: 3.

Figure 2 shows the graphs of SGN survival with the marked statistically detected differences of the Cometin (Figure 2A) and the EPO (Figure 2B) experiments. Mean and SEM of the detected survival rates are summarized in Table 3. In both series of experiments, the survival rate was significantly increased by the addition of 50 ng/mL BDNF as a positive control compared to the untreated NC (Cometin: *p* < 0.001; EPO: *p* < 0.01).

**Table 3:** Mean and Standard Error of Mean (SEM) of SGN survival rate and neurite length in experiments with Cometin and EPO.

*Survival rate*						
Cometin	Mean	± SEM		EPO	Mean	± SEM
NC	3.83	0.7		NC	7.30	0.54
PC (BDNF)	10.76	1.99		PC (BDNF)	11.40	0.77
HC10	12.86	1.21		HE10	1.63	0.26
MC10	4.92	0.70		ME10	6.23	0.65
HC1	5.38	0.59		HE1	8.01	0.98
MC1	4.08	0.68		ME1	5.54	0.95
HC0.1	3.62	0.30		HE0.1	7.15	0.64
MC0.1	3.04	0.44		ME0.1	6.46	0.62

*Neurite length*						
Cometin	Mean	± SEM		EPO	Mean	± SEM
NC	294.1	13.88		NC	338.4	12.27
PC (BDNF)	341.2	19.20		PC (BDNF)	379.7	10.94
HC10	296.3	10.72		HE10	218.1	17.40
MC10	275.5	11.95		ME10	285.4	12.72
HC1	275.6	13.76		HE1	318.4	11.15
MC1	271.3	15.46		ME1	243.6	10.05
HC0.1	240.1	11.20		HE0.1	320.2	12.20
MC0.1	244.9	13.77		ME0.1	276.0	10.67

The addition of 10 μg/mL human Cometin resulted in the highest detected survival rate of SGN compared to all other conditions. This increase in SGN survival was significant compared to the NC (*p* < 0.001), the lower concentrations of 1 μg/mL and 0.1 μg/mL human Cometin (HC1, HC0.1: *p* < 0.001) and the 10 μg/mL, 1 μg/mL, and 0.1 μg/mL concentrations of mouse Cometin (MC10, MC1, MC0.1: *p* < 0.001). Compared to the PC, 10 μg/mL human Cometin performed as well as BDNF in supporting the neuronal survival of SGN. With the exception of 10 μg/mL human Cometin, none of the lower human or any of the mouse Cometin concentrations had a significant effect on the neuronal survival compared to the NC, and survival was significantly lower than in the PC. A within species comparison indicated that only 10 μg/mL human Cometin performed better than the equivalent mouse concentration. This was also the case for comparison of the tested three concentrations of human and mouse Cometin.

In the EPO experiments, none of the tested mouse concentrations had a significant effect on SGN survival when compared to NC. Human EPO did not differ from NC when adding 0.1 ng/mL or 1 ng/mL to the SGN. Moreover, the lowest detected survival rate compared to NC (*p* < 0.001), PC (*p* < 0.001), and to all other EPO conditions (ME10, HE1, HE0.1, ME0.1: *p* < 0.001; ME1: *p* < 0.01) occurred after the addition of 10 ng/mL human EPO. All added EPO concentrations, whether human or mouse, resulted in significantly lower survival rates than that observed in the PC. Overall, a comparison between the two species and the three concentrations within species revealed that only the 10 ng/mL concentration of human EPO significantly reduced SGN survival.

### 3.2. Neuronal morphology

Neuronal morphology was assessed for each well by dividing the neurons into bipolar, monopolar, multipolar, pseudomonopolar, or neurons with no neurites. Figure 1 depicts representative neurons of each morphology.

**Figure 3:**
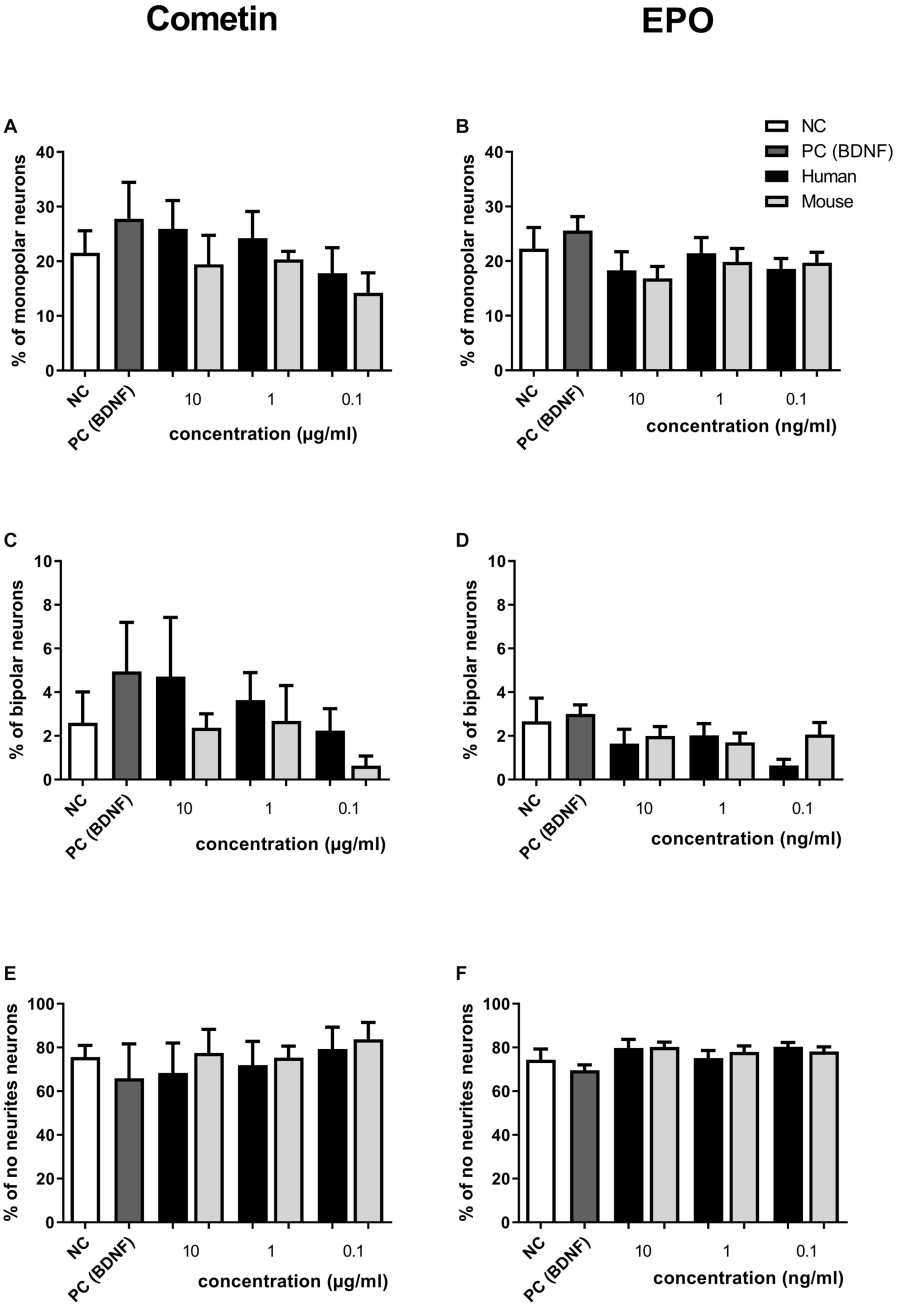
Influence of human and mouse Cometin and EPO on neuronal morphology after 48 h of incubation. SGN were distinguished into monopolar **(A,B)**, bipolar **(C,D)** and neurons without neurites **(E,F)** after treatment with Cometin **(A,C,E)** or EPO **(B,D,F)**. No relevant differences were detected between treatment conditions. Independent experiments: 3, wells per experiment: 3.

The pseudomonopolar and the multipolar neurons represent only a small percentage of the neuronal population (both below 5%) in Cometin and EPO conditions. Therefore, only the percentage of monopolar, bipolar and no neurite neurons are presented in Figure 3. Irrespective of the treatment condition, neurons without neurites were most frequently observed followed by monopolar and bipolar neurons. No statistically relevant differences in neuron morphology between applied factor concentrations, factor origin or controls were detected.

### 3.3. Neurite outgrowth

**Figure 4:**
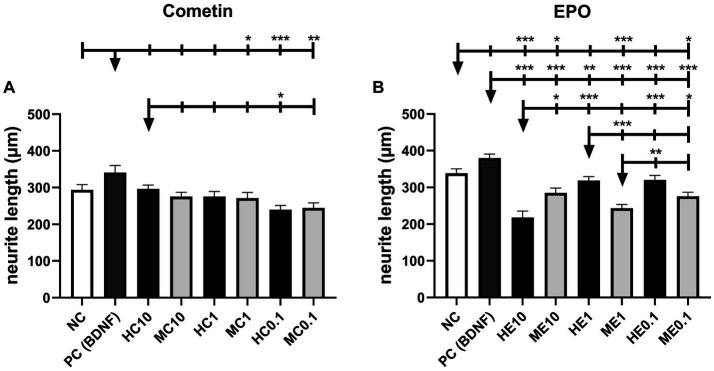
Neurite length of Cometin **(A)** and EPO **(B)** treated neurons. Neither Cometin nor EPO treatment increased neurite outgrowth compared to the relevant NC. Compared to PC, neurites were shorter for SGN treated with 1 μg/mL mouse Cometin and 0.1 μg/mL mouse and human Cometin. Treatment with 0.1 μg/mL human Cometin mediated the shortest detected neurite length overall. In the case of EPO, neurites were significantly shorter than in PC for all treatments and compared to NC for 10 ng/mL human, and 10 ng/mL, 1 ng/mL, and 0.1 ng/mL mouse EPO. Within the EPO-treated groups, neurites were shortest for 10 ng/mL human EPO. The lower concentrations of human EPO and all concentrations of mouse EPO did not differ from each other in their neurite length. At the same concentrations, 10 ng/mL human EPO resulted in shorter, 1 ng/mL human EPO in longer, and 0.1 ng/mL human EPO in an equivalent neurite length compared to mouse EPO. Asterisks above the error bars indicate the significance of the condition compared to the respective condition indicated by the arrowhead. ***p < 0.001, **p < 0.01, *p < 0.05; independent experiments: 3, wells per experiment: 3.

Measured neurite lengths of Cometin and EPO experiments are illustrated in Figure 4 (Cometin: Figure 4A; EPO: Figure 4B) and include detected significant differences. Mean and SEM are summarized in Table 3. In both experimental sets, longest neurites were detected for BDNF-treated SGN in the PC, although this difference was not significant compared to the untreated NC.

Cometin treatment had no significant effect on the length of outgrowing neurites compared to NC, regardless of species origin or concentration. Neurites were significantly shorter than in the PC for SGN treated with 1 μg/mL mouse Cometin (MC1: *p* < 0.05) and 0.1 μg/mL human and mouse Cometin (HC0.1: *p* < 0.001; MC0.1: *p* < 0.01). For 10 μg/mL human and mouse Cometin and 1 μg/mL human Cometin, neurite length did not differ significantly from PC. Overall, the shortest neurites were measured for 0.1 μg/mL human Cometin. This length was significantly shorter than in PC and for10 μg/ml human Cometin (PC: p < 0.001, HC0.1: p < 0.05). Except for 0.1 μg/mL human Cometin, no differences were observed within the mouse and human Cometin groups or between the three tested concentrations.

In the EPO experiments, neurite outgrowth was significantly shorter for neurons treated with the different concentrations of mouse EPO when compared to NC (ME10, ME0.1: p < 0.05; ME1: p < 0.001). SGN provided with 1 ng/mL or 0.1 ng/mL human EPO regenerated neurites in a length not differing significantly from NC, while 10 ng/mL human EPO induced significantly shorter neurite outgrowth (HE10: *p* < 0.001). Compared to PC, all EPO-treated groups resulted in significantly shorter neurites, regardless of species source and concentration (HE10, ME10, ME1, HE0.1, ME0.1: *p* < 0.001; HE1: *p* < 0.01). Neurite outgrowth was overall significantly shortest for 10 ng/mL human EPO (ME10, ME0.1: *p* < 0.05; HE1, HE0.1: *p* < 0.001) with the exception of 1 ng/mL mouse EPO. Within the human EPO concentrations tested, 10 ng/mL resulted in the shortest neurites, while there was no difference in neurite length between 1 ng/mL and 0.1 ng/mL EPO. The three tested concentrations of mouse EPO had no significant different effect on the neurite length. Comparing human with mouse EPO at the same concentrations, neurites were longer for 10 ng/mL mouse EPO (ME10: *p* < 0.05), while in case of 1 ng/mL neurites were longer for human EPO (ME1: *p* < 0.001). There was no detectable difference between species for the 0.1 ng/mL concentration.

## 4. Discussion

Protection of SGN from degeneration is a major topic in hearing research since their number and vitality are regarded to be a prerequisite for successful speech perception with a CI (Jelkmann, 2016; Cheng and Svirsky, 2021). Additionally, their functional integrity is a fundamental requirement to help establish future therapeutic strategies such as multi-electrode inner ear implants and hair cell regenerating approaches. An impressive body of experimental work has been performed to date investigating the effects of NTF application on SGN. However, treatment with some NTF has yielded contradictory results within the performed studies. We hypothesize that these differences in efficacy may be due to the species origin and homology of the tested proteins used. The aim of the work presented here was to investigate if the origin of the factors – mouse or human – influences the results of neuronal survival, morphology or neurite length of the primary auditory neurons, the spiral ganglion neurons *in vitro*.

Experiments were performed in two timely independent test series, one for Cometin and one for EPO, each of which incorporated 3 independent experiments with 3 wells per condition. Cometin and EPO results were considered separately and not compared with each other since the aim of the study was comparing mouse vs. human factors.

The concentration ranges of mouse and human Cometin tested in the current study were chosen based on previous findings. Accordingly, recombinant human Cometin has been reported to significantly induce STAT3 phosphorylation in E16-E18 embryonic rat cortical neurons at 5 μg/mL (R&D Systems, 2023a). Importantly, mouse recombinant Cometin in the range of 20–100 ng/μL, significantly enhances neurite outgrowth of E16-E18 rat embryonic cortical neurons (R&D Systems, 2023b), while administration at 1 μg/mL supports survival of SGN *in vivo* (Jørgensen et al., 2012). On the other hand, much lower concentrations (5 ng/mL) of recombinant human EPO resulted in increased neurite length *in vitro* (Berkingali et al., 2008). Therefore, Cometin was chosen in a μg/ml range whereas EPO was applied in a ng/ml range. Human and mouse Cometin were tested at three different concentrations: 0.1, 1, and 10 μg/mL. 10 μg/mL human Cometin significantly increased the neuronal survival compared to the relevant NC and this effect did not differ from that of the established positive control BDNF. This positive effect on SGN survival was significant in comparison to the mouse Cometin used in the same concentration and to all other Cometin concentrations, whether mouse or human. For Cometin, it seems as if the highest tested concentration is most beneficial for SGN survival when it is of human origin but not of mouse. We think it is noticeable that there might be a dose-related increase of SGN survival (Figure 2A) and neurite outgrowth (Figures 3A,C,E, 4A) for human or mouse Cometin, from 0.1 to 10 μg/mL. Such a dose-related response is already described by Jørgensen who applied mouse Cometin to dorsal root ganglions *in vitro* and detected longer neurites with increasing Cometin concentrations (Jørgensen et al., 2012). Based on the presented data in the current study, it can be assumed that higher concentrations of human Cometin could possibly induce even more pronounced effects. Such dose dependency may be involved in the biological actions of mouse Cometin on SGN as well. However, we cannot exclude that the tested concentrations in the current study were too low and that with increasing mouse Cometin concentrations higher SGN survival rates might have been achieved. Increased human and mouse concentrations should therefore be investigated in future studies before assessing this growth factor in *in vivo* application.

While the highest tested human Cometin concentration of 10 μg/mL significantly increased neuronal survival, in contrast the highest human EPO concentration of 10 ng/mL significantly decreased SGN survival compared to the NC (Figure 2B). None of the lower EPO or Cometin concentrations nor the 10 μg/mL mouse Cometin and 10 ng/mL mouse EPO differed from the NC, indicating that EPO, no matter if from human or mouse source, does not have a positive effect on SGN. All EPO conditions resulted in significantly decreased SGN survival compared to the BDNF condition. The neurite outgrowth of EPO treated SGN regardless of species origin and concentration was not increased compared to NC or was even significantly reduced in case of the tested mouse EPO concentration and 10 ng/mL human EPO (Figures 3B,D,F, 4B). When excluding the human EPO 10 ng/mL condition, the lower human EPO concentrations did not reduce regenerated neurite length in a similar manner to mouse EPO.

A wide number of experimental studies have shown that EPO causes remarkably beneficial effects in the hearing system (Andreeva et al., 2006; Monge et al., 2006; Quesnel et al., 2011; Gürgen et al., 2014). It is stated that it has a selective effect on neurite outgrowth of SGN rather than on neuronal survival in the rat (Berkingali et al., 2008). Our data show that EPO has no protective effects on SGN and shows no beneficial effect on neurite length when human or mouse EPO is used. This is, for human EPO, consistent with previously published work (Kaiser et al., 2013) where unfortunately the species origin of EPO was not stated. The initial hypothesis that the EPO source may be relevant for its beneficial biological effect is not supported by our data. Nevertheless, although the selected concentration range was chosen based on published data, we cannot exclude that the missing protective effect or the detected negative effect of 10 ng/mL human and, regarding the neurite length, for mouse EPO in our study is based on too low or too high concentrations.

In contrast, for Cometin our hypothesis seems to be supported by the data. Human Cometin was clearly more effective at preserving rat SGN from degeneration and inducing neurite outgrowth than mouse Cometin. This is a very interesting finding, as one would expect a better effect of mouse protein on the rat SGN since the amino acid sequence of mouse and rat Cometin precursors has a greater consistency than the human one. A reason for the higher efficacy of human Cometin on rat cells could be that in some cases human and rat, but not mouse, share the same amino acid in the precursor sequence (Zheng et al., 2016). However, mouse and human Cometin are 77% identical proteins of 311 amino acids with a predicted signal peptide and apparently no pro-sequence, resulting in a 266 amino acid mature protein with a mass of 30 kDa. According to the NCBI database, Cometin is located on mouse chromosome 11qE2 whereas the human version is found on chromosome 17q25.3 (Jørgensen et al., 2012; Zheng et al., 2016). These differences may possibly account for variations in biological efficacy. For example, a single amino acid substitution in 5-Lipoxygenase Activating Protein (FLAP) is responsible for the specificity and differential pharmacology of novel FLAP inhibitors in mouse and human (Blevitt et al., 2016).

Recently, KIT receptor tyrosine kinase has been identified as a putative receptor for Cometin in cardiac tissue (Reboll et al., 2022). Intriguingly, a novel point mutation in the Kit gene in mice has been linked to a loss of function and a number of physiological deficits that include deafness (Ruan et al., 2005). Accordingly, transfection of a cell line such as HEK-293 cells with cDNAs encoding for murine and human KIT receptors could provide a comparison of Cometin binding to exclude this as an issue that might account for the potency/efficacy difference between mouse and human Cometin on SGN protection. However, this would still need to be linked to a reporter assay for a more extensive validation to confirm that any binding differences have functional consequences for downstream signaling events. This approach is far from trivial and at the time these experiments were performed, in the absence of the receptor, we proceeded with the latter approach.

Studies on drug effects on the inner ear often lack details such as correct naming of the compound (Salt and Plontke, 2020) or precise description of the protein used. In view of the known problem of differential pharmacology between species, or speciation encountered during the course of drug discovery, this lack of fundamental information in experimental reporting can severely hamper efficient translation from the lab to clinical application. Many examples of species specificity that have impacted drug discovery efforts exist in the literature and cover a broad range of protein families including G protein-coupled receptors (Tichenor et al., 2015), cytochrome P450 isoforms (Lewis et al., 2009), and the MAPEG member microsomal prostaglandin E synthase-1 (Xu et al., 2008). In the simplest of cases, research teams have identified an appropriate preclinical species, with homologous target protein sequence to human, to facilitate the optimal development of translational models. In more challenging settings, where this is not possible because of the lack of an acceptable preclinical species, the use of humanized mice may be required (Shultz et al., 2007).

For the development of inner ear pharmacotherapies, this work is the first to describe that species specificity of NTF can profoundly affect the biological outcome. Next to other confounds, such as age and sex, the species specificity of the tested active pharmaceutical ingredient needs to be adequately taken into account when choosing a protein for testing and it should be communicated in detail when data are published.

## 5. Conclusion

This study describes the protective effect of Cometin and EPO proteins from different species sources on auditory neurons *in vitro*. Human Cometin significantly improved SGN survival and clearly proved to be as beneficial as BDNF, and superior to mouse Cometin. Additionally, 10 μg/mL and 1 μg/mL human and 10 μg/mL mouse Cometin resulted in the same neurite length as detected for BDNF treated neurons. EPO has no beneficial effect on SGN survival regardless of mouse or human source, while neurite length was reduced by treatment with mouse EPO.

This study is the first reporting differences in the biological effect on auditory neurons of proteins generated from human or mouse sources. Using proteins from different species non-discriminatively without due consideration to their inherent properties may be one reason for the high variability of functional and histological outcomes of some inner ear pharmacotherapeutics.

## Data availability statement

The raw data supporting the conclusions of this article will be made available by the authors, without undue reservation.

## Ethics statement

The animal study was reviewed and approved by the local authorities of Hannover Medical School.

## Author contributions

VS, JS, GM, and KP conceived and designed the experiments. ZG and JS performed the experiments. VS, ZG, and JS analyzed the data. VS and TL contributed reagents, materials, and analysis tools. VS, ZG, JS, and TL wrote the paper. All authors have given approval to the final version of the manuscript.

## Funding

This study was funded by the Deutsche Forschungsgemeinschaft (DFG, German Research Foundation) under Germany’s Excellence Strategy – EXC 2177/1–Project ID 390895286. This publication is funded by the Deutsche Forschungsgemeinschaft (DFG) as part of the “Open Access Publikationskosten” program.

## Conflict of interest

Authors GM and KP were employed by company Hoba Therapeutics ApS.

All authors declare that this study received funding from Hoba Therapeutics ApS. This funder had the following involvement in the study: contribution of reagents and materials, study design, and preparation of the manuscript.

## Publisher’s note

All claims expressed in this article are solely those of the authors and do not necessarily represent those of their affiliated organizations, or those of the publisher, the editors and the reviewers. Any product that may be evaluated in this article, or claim that may be made by its manufacturer, is not guaranteed or endorsed by the publisher.

## Footnotes

^1^ https://www.olympus-lifescience.com

## Abbreviations

BDNF: brain-derived neurotrophic factor
CI: cochlear implant
DAB: 3,3′-Diaminobenzidine
EPO: erythropoietin
GDNF: glial-cell line derived neurotrophic factor
HC: human cometin
HE: human EPO
HBSS: hank’s balanced salt solution
MC: mouse cometin
ME: mouse EPO
NT-3: neurotrophin-3
NTF: neurotrophic factor
NC: negative control
PBS: phosphate-buffered saline
PC: positive control
SEM: standard error of the mean
SGC: spiral ganglion cells
SGN: spiral ganglion neurons
SNHL: sensorineural hearing loss
